# Obstructed Hemivagina and Ipsilateral Renal Anomaly (OHVIRA) Syndrome: A Case Report

**DOI:** 10.7759/cureus.101002

**Published:** 2026-01-07

**Authors:** Yin Ru Tan, Ravichandran Nadarajah, Hak Koon Tan

**Affiliations:** 1 Urogynecology, KK Women's and Children's Hospital, Singapore, SGP; 2 Gynecologic Oncology, KK Women’s and Children’s Hospital, Singapore, SGP; 3 Obstetrics and Gynecology, KK Women's and Children's Hospital, Singapore, SGP

**Keywords:** dysmenorrhoea, herlyn-werner-wunderlich syndrome, lower abdominal pain, müllerian duct anomalies, ohvira syndrome, unilateral renal agenesis

## Abstract

Obstructed hemivagina and ipsilateral renal anomaly (OHVIRA) syndrome is a rare congenital genitourinary tract anomaly characterized by the triad of uterine didelphys, unilateral obstructed hemivagina, and ipsilateral renal agenesis. Patients typically present with symptoms of cyclical pain after menarche. Cases are usually complex and may pose difficulties in diagnosis and management. Accurate diagnosis and prompt treatment are essential to relieve patient symptoms and prevent complications. We present a case of OHVIRA syndrome that was successfully managed surgically.

## Introduction

Müllerian duct anomalies are complex anomalies of the female genital tract that can affect the uterus, cervix, and vagina [1]. They affect 5.5% of the general population [2]. Obstructed hemivagina and ipsilateral renal anomaly (OHVIRA) syndrome, also known as Herlyn-Werner-Wunderlich syndrome, is a rare complex congenital genitourinary tract anomaly characterized by the triad of uterine didelphys, unilateral obstructed hemivagina, and ipsilateral renal agenesis [1]. Patients typically present with symptoms of cyclical pain after menarche.

We present a case of a 15-year-old female patient with OHVIRA syndrome, who was successfully treated with laparoscopy. Written informed consent was obtained from the patient.

This case was previously presented as a poster at the 32nd World Congress on Controversies in Obstetrics, Gynecology & Infertility (COGI) on November 21, 2024.

## Case presentation

**Figure 1:**
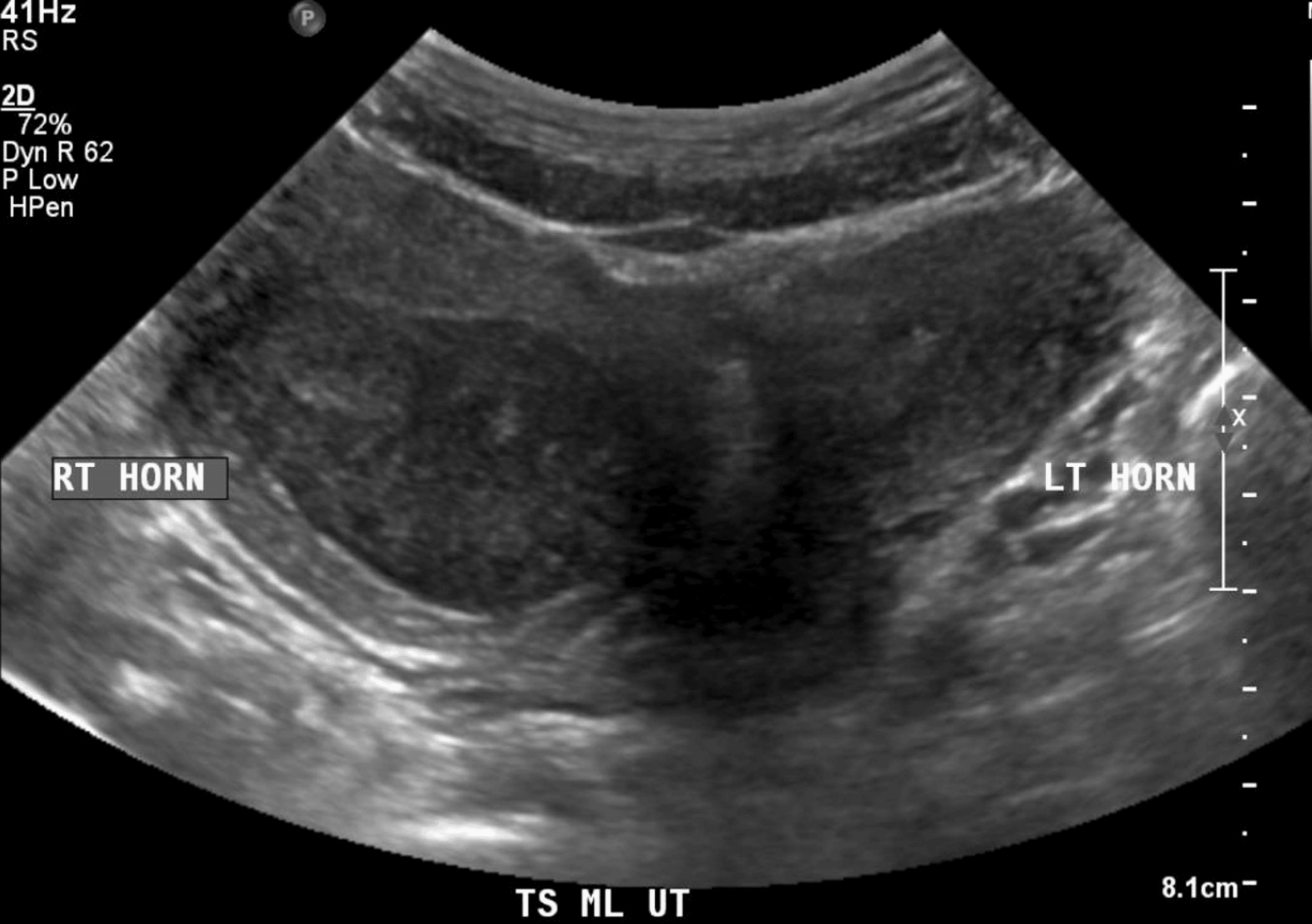
Transverse view of the uterus on transabdominal ultrasound showing uterine didelphys

The patient was a 15-year-old female with no significant past medical or surgical history. She presented with a three-year history of dysmenorrhea and cyclical lower abdominal pain, which was affecting her daily routine. Her menarche was at 10 years of age, and she had regular monthly periods. She had dysmenorrhea every month, but her abdominal pain became progressively severe, requiring increasing doses of analgesics. Blood investigations were unremarkable. Clinical examination revealed lower abdominal tenderness with a palpable right lower abdominal mass. Her introitus appeared normal. A transabdominal ultrasound of the pelvis showed uterine didelphys (Figure 1).

**Figure 2:**
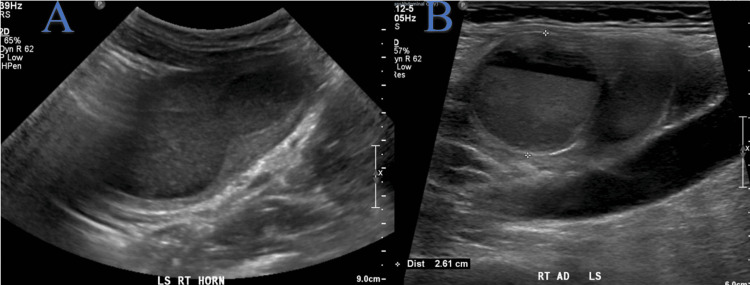
Longitudinal view of transabdominal ultrasound showing (A) right uterine horn with right hematometra and (B) right hematosalpinx

The right uterine corpus was grossly enlarged with the endometrial cavity distended with fluid suggestive of hematometra (Figure 2A). A convoluted tubular cystic structure containing fluid up to 2.6 cm in diameter suggesting a hematosalpinx was also noted in the right adnexa (Figure 2B).

Both ovaries were normal. An ultrasound of the kidneys showed the absence of the right kidney. The left kidney was normal, with no hydronephrosis noted.

**Figure 3:**
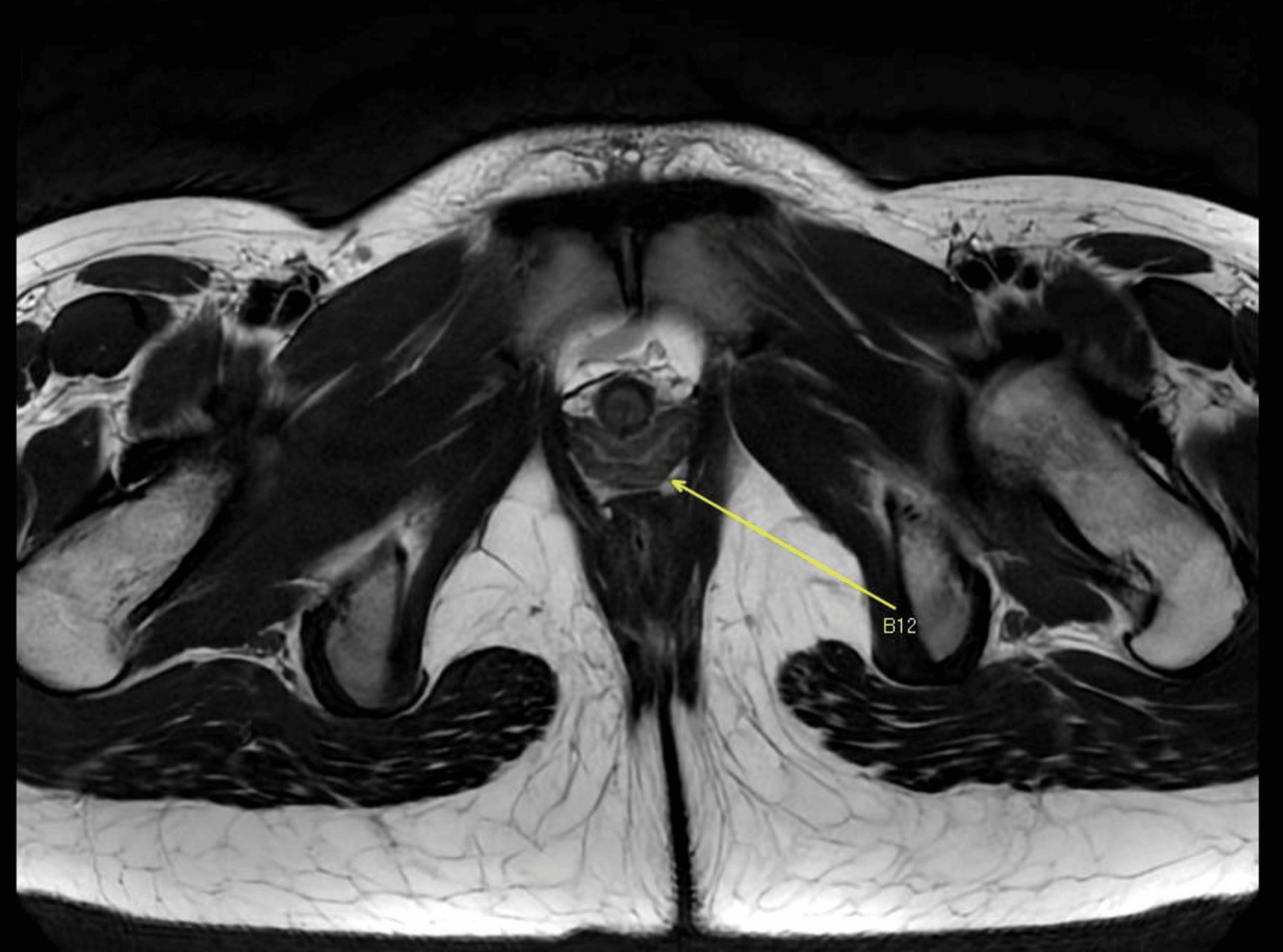
Axial view on MRI showing uterine didelphys and a common vaginal canal MRI: magnetic resonance imaging

Magnetic resonance imaging (MRI) of the pelvis was performed. The MRI pelvis showed uterine didelphys, with the two separate uterine horns communicating with a common vaginal canal (Figure 3).

**Figure 4:**
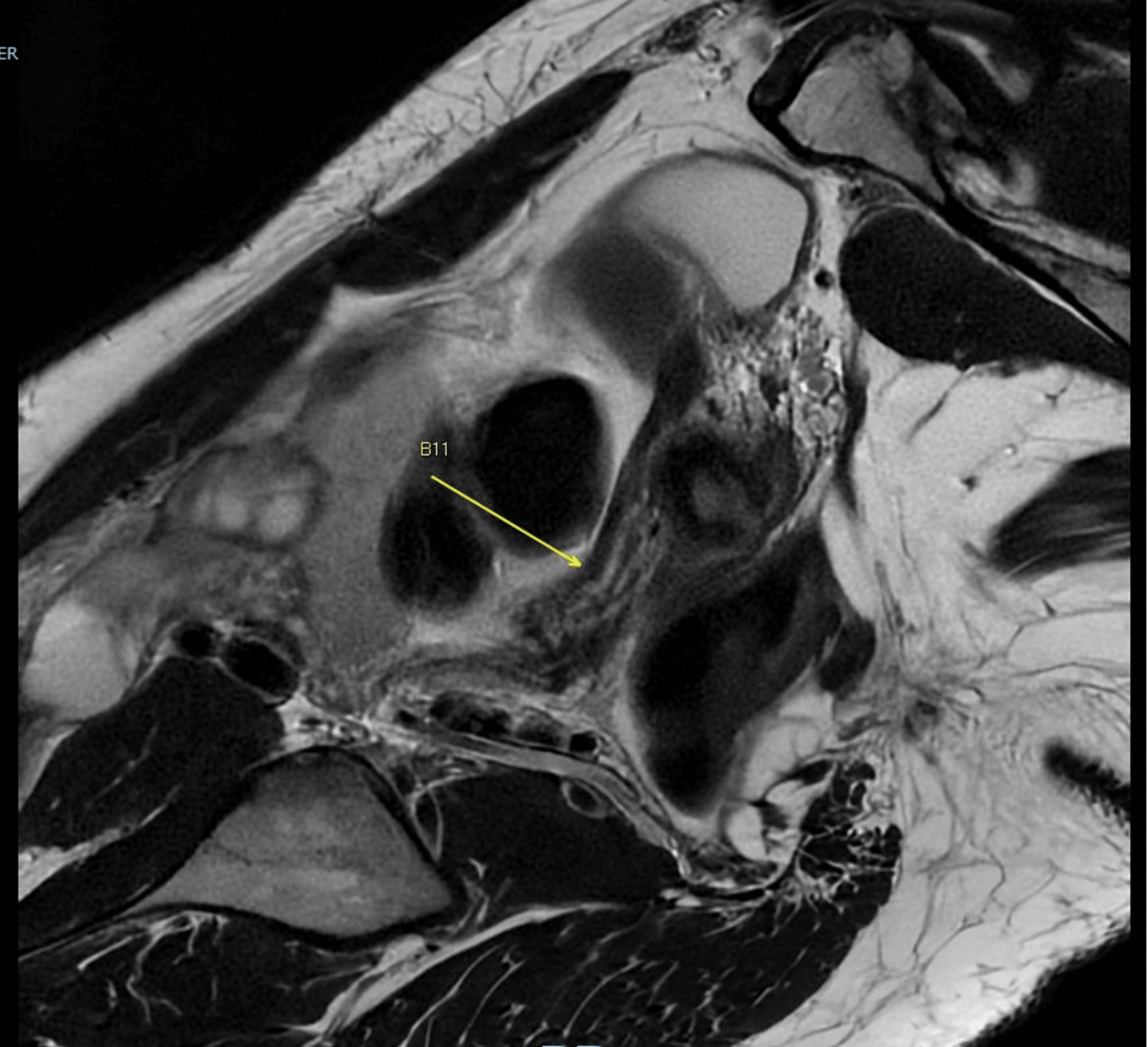
MRI image showing the right uterine horn communicating with a severely narrow right cervix MRI: magnetic resonance imaging

The right uterine horn appeared to be connected by a narrow ridge of soft tissue to a severely atretic right cervix (Figure 4).

**Figure 5:**
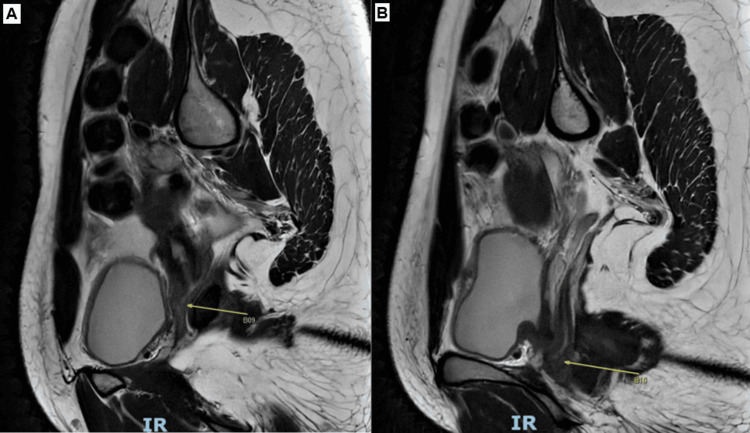
(A, B) MRI image showing the left uterine horn communicating with the left cervix and vagina MRI: magnetic resonance imaging

There was normal communication of the left uterine horn to the left cervix and the vagina (Figure 5).

**Figure 6:**
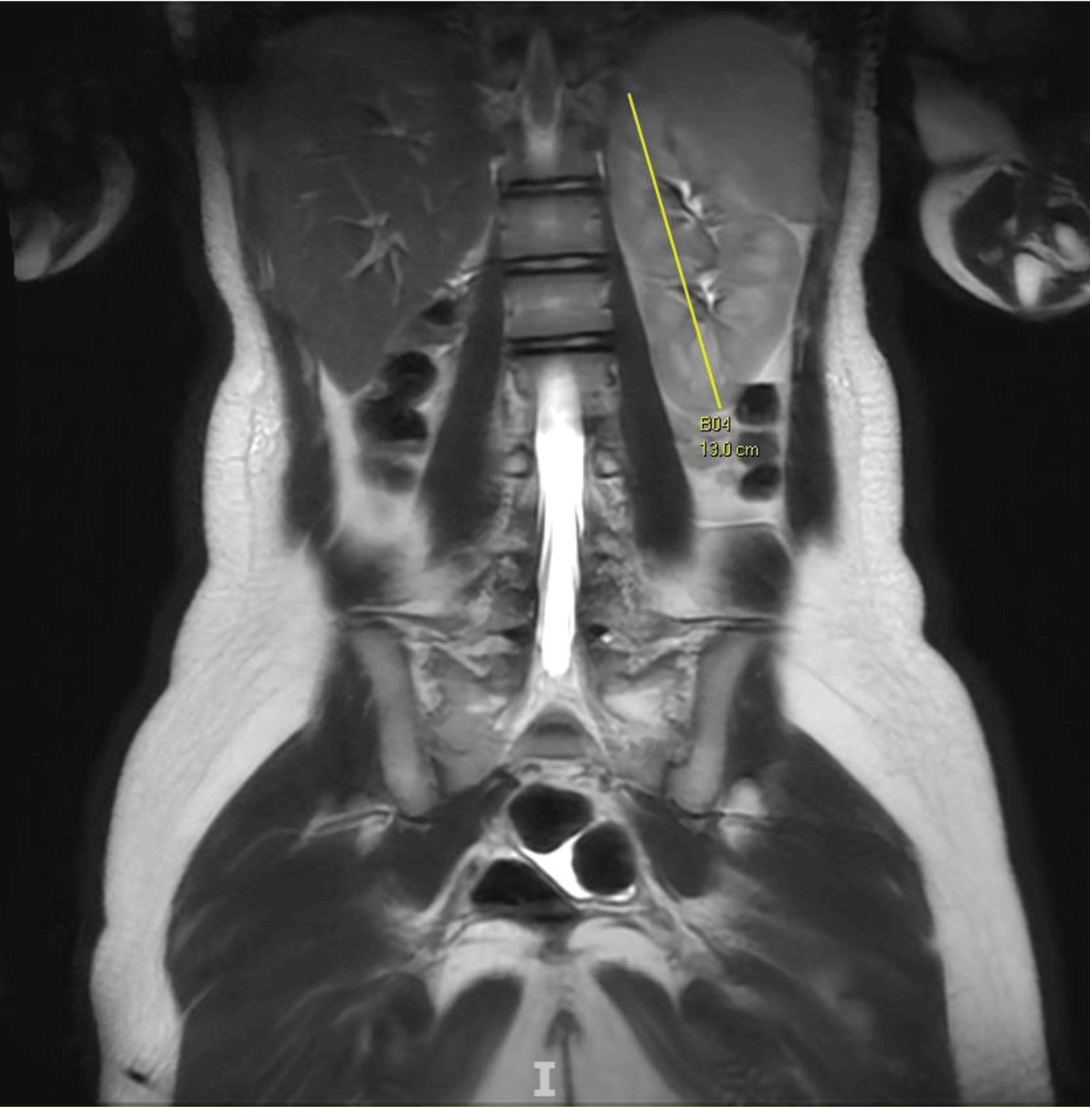
Coronal view on MRI showing right renal agenesis and left solitary kidney MRI: magnetic resonance imaging

An elongated structure in the right adnexa suggestive of hematosalpinx was seen. Both ovaries appeared normal. Right renal agenesis was noted, and the left solitary kidney appeared hypertrophic (Figure 6).

There was no hydroureteronephrosis or renal mass. These findings were suggestive of a variant of OHVIRA syndrome.

**Figure 7:**
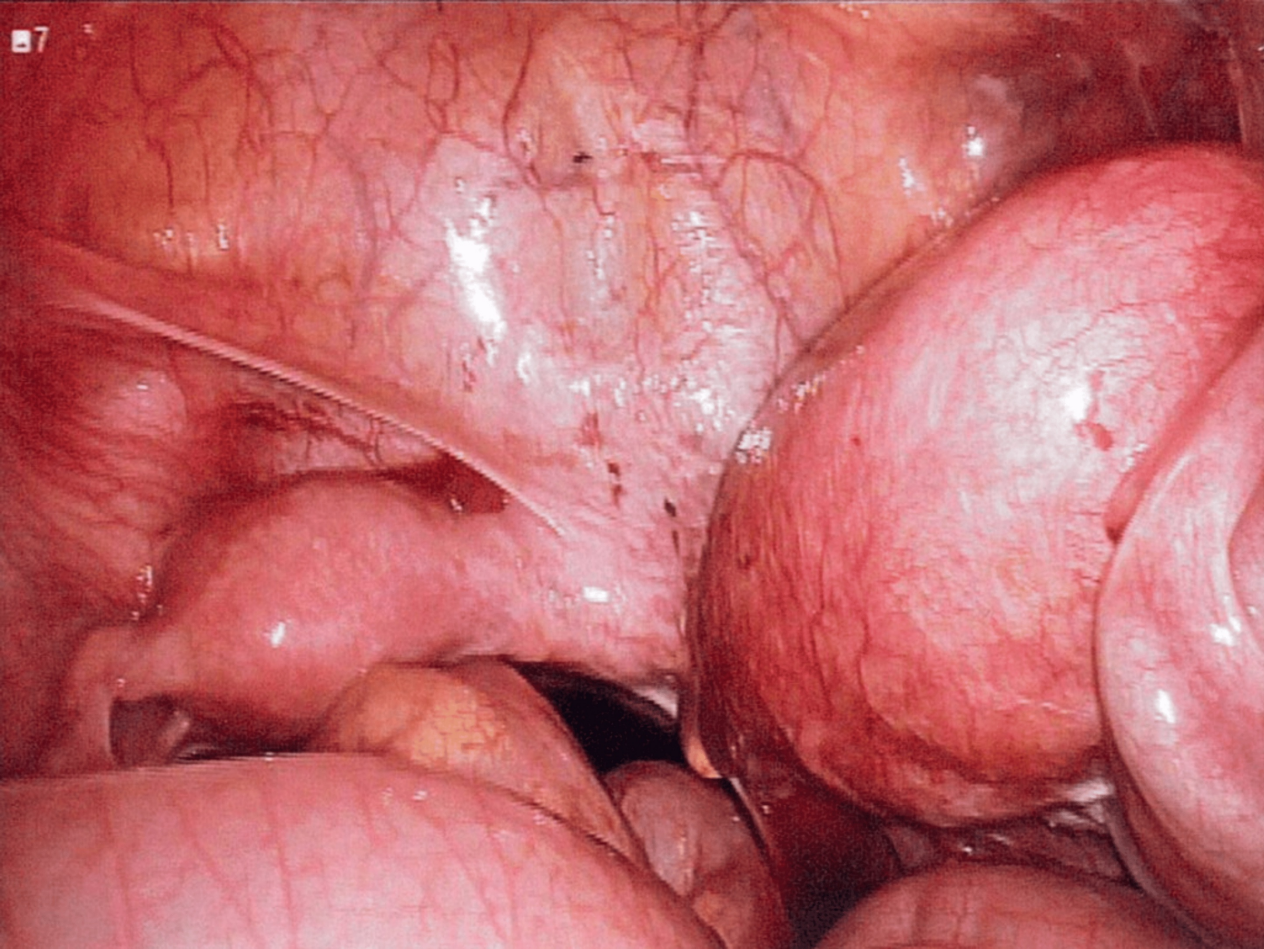
Laparoscopic image of the enlarged right uterus and right hematosalpinx intraoperatively

The patient underwent a diagnostic laparoscopy, examination under anesthesia, with excision of the right uterus and fallopian tube. Intraoperatively, a normal vagina with no hematocolpos or vaginal septum was seen. A single normal cervix was noted on vaginal examination. Diagnostic laparoscopy showed a normal left uterine horn and fallopian tube. A single left cervix was connected to the left uterus. The right uterine horn was enlarged up to 14 weeks in size with hematometra, and a right hematosalpinx was seen (Figure 7).

**Figure 8:**
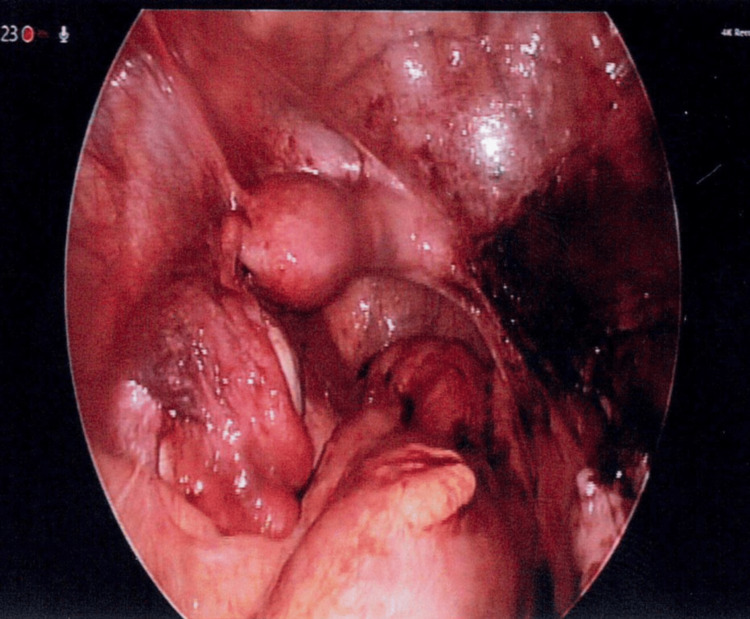
Laparoscopic image after the right uterine horn and fallopian tube excised

A normal right cervix was absent. The right uterine horn appeared to be connected by a narrow ridge of soft tissue to the left cervix. Hemosiderin stains were noted on the omentum and peritoneum. The abnormal right uterus and fallopian tube were excised and removed via single-port laparoscopy (Figure 8).

Histopathological examination of the excised right uterus and fallopian tube showed proliferative endometrium and features consistent with cervical endometriosis. Tubal endometriosis with chronic salpingitis and hydrosalpinx was also noted. There was no malignancy.

The patient recovered well after the procedure and was discharged on postoperative day 3. She was well up to 12 months after surgery. Her periods were regular monthly, and her symptoms of cyclical abdominal pain resolved.

## Discussion

OHVIRA syndrome is a rare complex Müllerian anomaly with a reported incidence of 0.1%-3.8% [3]. Patients commonly present with cyclical abdominal pain soon after menarche due to hematocolpos or hematometra [3]. However, as patients may have normal periods from the normal unobstructed hemivagina, their condition may be misdiagnosed and diagnosis may be delayed. A recent retrospective study found that 16.5% of patients with OHVIRA syndrome were misdiagnosed [4]. If untreated, complications such as hematosalpinx, endometriosis, pelvic inflammatory disease, pelvic adhesions, or infertility may occur [2,5]. Therefore, early diagnosis and treatment of OHVIRA syndrome are crucial.

There is a wide range of anatomical variants and clinical manifestations of Müllerian anomalies and OHVIRA syndrome [4]. Müllerian anomalies are commonly classified according to the American Society for Reproductive Medicine (ASRM) Müllerian anomalies classification [1] or the European Society of Human Reproduction and Embryology (ESHRE)/European Society for Gynecological Endoscopy (ESGE) consensus on the classification of female genital tract congenital anomalies [6]. Zhu et al. classified OHVIRA syndrome into two main categories based on whether the hemivagina was completely obstructed [7]. This is an interesting case of a variant of OHVIRA syndrome with cervical atresia where the hemivagina was obstructed.

Diagnosis of OHVIRA syndrome is challenging. Established imaging modalities include pelvic ultrasound and MRI [8,9]. MRI is currently the gold standard imaging modality for the evaluation of OHVIRA syndrome [9-11]. Increased availability of MRI has led to improvements in the diagnosis and management of OHVIRA syndrome. For our patient, both pelvic ultrasound and MRI were performed for diagnosis and to aid in preoperative planning.

The treatment of OHVIRA syndrome is usually surgical, although temporary medical management for menstrual suppression can be considered till surgery can be performed [10,12]. Surgical management typically involves excision of the vaginal septum with drainage of hematocolpos or hematometra [10,13]. Non-communicating rudimentary horns, which may cause symptoms, are usually resected [10]. In this case, we had to remove the right uterine horn, which was not communicating with the vagina.

## Conclusions

OHVIRA syndrome is a rare condition. This case demonstrates the typical presentation, established imaging modalities, and successful surgical management of a patient with OHVIRA syndrome. Accurate diagnosis and prompt treatment of OHVIRA syndrome are essential to relieve patient symptoms and prevent complications.
